# Guided Growth for the Treatment of Cubitus Varus in Children: Medium- to Long-Term Results

**DOI:** 10.3390/jcm12072632

**Published:** 2023-03-31

**Authors:** Sergio Martínez-Álvarez, María Galán-Olleros, Javier Alonso-Hernández, Isabel Vara-Patudo, Carlos Miranda-Gorozarri, Ángel Palazón-Quevedo

**Affiliations:** Pediatric Orthopaedics, Orthopaedic Surgery and Traumatology Department, Hospital Infantil Universitario Niño Jesús, Av. de Menéndez Pelayo 65, 28009 Madrid, Spain

**Keywords:** cubitus varus, gunstock deformity, elbow, pediatrics, hemiepiphysiodesis, humeral physis, supracondylar fracture, guided growth, satisfaction

## Abstract

Correction of cubitus varus is commonly attempted through supracondylar humeral osteotomy. We hypothesized that lateral distal humeral hemiepiphysiodesis (LDHH) could be used to gradually correct this deformity in children. We conducted a retrospective study including all patients who underwent LDHH with the eight-Plate system between 2008 and 2018, with a minimum 4-year follow-up. We collected demographic, fracture-related, pre- and postoperative clinical (carrying angle (CA), ROM), and radiological data (humeral-ulnar angle (HUA), Baumann angle (BA), shaft-condylar angle (SCA), lateral capitellohumeral angle (LCHA)), as well as data on complications and satisfaction at last follow-up. Fifteen patients were included, with a median follow-up of 81 (64–103) months. All the variables had improved significantly as follows: CA −16 (−18 to −9)°, HUA −16 (−19 to −12)°, BA −11 (−17 to −7)°, SCA 7.5 (3.3 to 13.8)°, LCHA −4.8 (−6.8 to 0.6), flexion 10 (0 to 24)°, and extension 10 (0 to 10)°. The annual correction rate in terms of HUA was 2.41° (1.9 to 3.2). There were 5 cases of aseptic screw loosening, 4 of them requiring replacement, without relation to age at surgery (*p* = 0.324). Most patients (86.67%) were satisfied, and a relationship was found with younger age at surgery (*p* = 0.037). In conclusion, preliminary results show that LDHH with the eight-Plate system is an effective technique for mild to moderate cubitus varus deformity correction in children. Patients should be advised of the relatively long duration of implant retention and the possibility of reoperation for screw replacement or implant removal.

## 1. Introduction

Cubitus varus is the most common elbow deformity secondary to elbow fracture in children. In addition to the cosmetic appearance (gunstock deformity) caused by this deformity, patients with cubitus varus may develop chronic pain, early osteoarthritis [1], ulnar neuropathy [2], posterolateral rotatory instability (PLRI) of the elbow [3], or triceps snapping [4] over the long term [5].

Correction of cubitus varus is commonly attempted through supracondylar humeral osteotomy. There are numerous publications evaluating the results of supracondylar osteotomy of the humerus to correct the deformity. Although studies comparing different types of osteotomies have been published, there remains no evidence indicating which is best [6,7,8,9]. This is a technically demanding procedure requiring great precision to properly align the humerus [10]. For this reason, a number of surgical assistance methods have been designed in recent years to improve the accuracy of osteotomy and fixation [11,12,13,14], although their use is not as widespread as expected and their cost is high. In addition, as osteotomy aims to correct the deformity in a single surgical event, these interventions carry a risk of nerve injury [6] and require postoperative immobilization.

As in other angular deformities of the extremities, guided growth has been proposed as a minimally invasive technique that allows gradual correction in children [15]. Guided growth has the advantage of allowing follow-up over time until there is evidence of complete correction and, as a progressive intervention, is assumed to avoid the neurological complications associated with acute corrections. Furthermore, guided growth is less invasive and does not require prolonged immobilization as with corrective osteotomy. However, few recent studies have analyzed this technique applied to the distal humerus for the correction of cubitus varus deformity in children, obtaining uneven results among them [16,17,18].

We hypothesized that lateral distal humeral hemiepiphysiodesis (LDHH) can be used for the gradual correction of cubitus varus deformity in children. The aims of this study are to describe the surgical technique of LDHH with the eight-Plate system used in our pediatric referral center to evaluate the medium-term results and complications of the technique in a group of pediatric patients, and to detect the factors associated with a higher degree of correction and greater satisfaction.

## 2. Materials and Methods

Following institutional review board approval (NO. R-0070-22), a retrospective study was performed in a pediatric referral center between 2008 and 2018, including all patients who underwent LDHH for cubitus varus as a sequela of distal humerus fracture. The main indications for surgery were deformity progression, cosmetic unacceptability, or long-term sequelae concerns by parents. Patients with previous corrective osteotomy, neurovascular injuries, open fractures, and elbow stiffness were excluded. A minimum follow-up of 4 years was required to enable proper evaluation of the results of this technique, which is based on gradual correction throughout the growth period.

**Figure 1:**
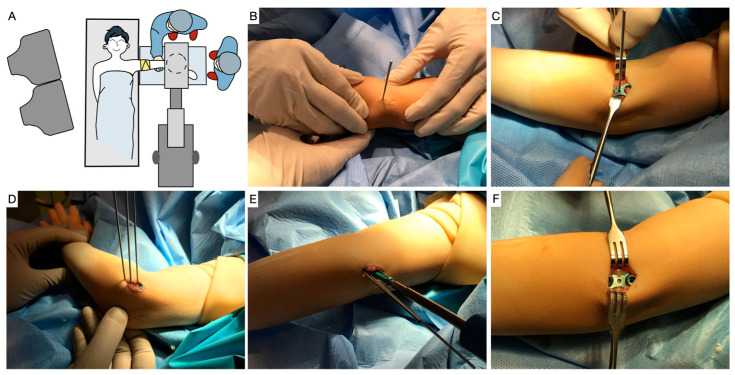
(**A**) Operating room layout with the patient in a supine position and the affected arm on radiolucent hand table. The patient’s shoulder is centered, in 90° of abduction and internal rotation and the elbow is flexed at 60–70° with the forearm in pronation. The image intensifier is set up from the bottom and screens are placed in front of the surgeon. The tourniquet is placed on the upper arm with a sterile Esmarch bandage. (**B**) Guide wire located in the lateral humeral condyle physis under fluoroscopic guidance and 2 cm incision centered over the guide wire. (**C**,**D**). Placement of eight-Plate and two more guide wires, proximal and distal. (**E**) Cannulated screw placement. (**F**) Intraoperative result after placement of the eight-Plate and screws in the lateral distal humerus.

In our institution, the procedure is performed under general anesthesia, with the patient supine and the arm placed on a radiolucent hand table. Prophylactic antibiotics are administered prior to the application of a sterile tourniquet on the arm. The physis of the lateral humeral condyle is located using a guide wire under fluoroscopic guidance. A 2 cm incision is made in the lateral aspect of the distal humerus, centered over the guide wire (Figure 1). The subcutaneous tissue and the muscular layer between the brachialis anterior and the triceps brachii posteriorly are dissected until the periosteum is reached, without opening it.

**Figure 2:**
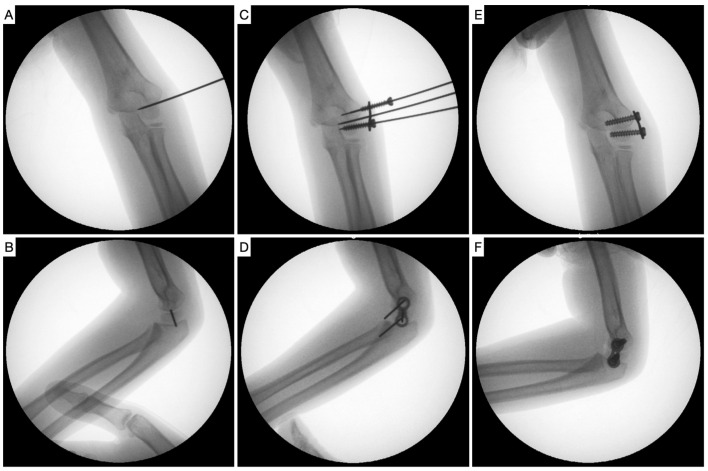
(**A**,**B**) Guide wire placement in the lateral humeral condyle physis centered in both planes. (**C**,**D**) Placement of the eight-Plate, guide wires, and proximal and distal screws in both projections. (**E**,**F**) Final check of correct position of the eight-Plate and screws.

Once the periosteum is reached, correct positioning of the central guide wire in the physis is checked with fluoroscopy, and the eight-Plate^TM^ guided growth system (Orthofix Srl, Verona, Italy) is placed and held with two more guide wires, located proximally and distally. After further fluoroscopic verification of correct positioning in both planes, two 4.5 mm canulated screws are placed, one penetrating the capitellar epiphysis and the other over the humeral metaphysis, avoiding penetration into the olecranon fossa (Figure 2). In very young children, with little ossification of the elbow, arthrography is used to facilitate the correct position and avoid intra-articular penetration of the screws. If there is no sagittal plane deformity, the plate is placed following the sagittal orientation of the distal humerus, with a certain anterior obliquity, while when there is a hyperextension deformity, an attempt is made to place the plate in the most anterior area of the condyle.

After the intervention, a compression bandage is placed to allow mobility of the elbow and the patient is discharged on the first postoperative day. Plain radiographs are taken in the anteroposterior and lateral views of the elbow, and clinical evaluations are performed at 1, 6, and 12 months postoperatively, and subsequently every 1 or 2 years depending on the patient’s growing period. Plates are usually maintained until correction of the deformity or until skeletal maturity.

We collected data on demographics (age at the time of LDHH surgery, gender, affected side) as well as fracture-related data (type of fracture that caused cubitus varus, treatment modality). Pre- and postoperative clinical data (carrying angle (CA), range of movement (ROM), PLRI of the elbow, and signs or symptoms of neuropathy) as well as radiological findings and surgery-related data were also recorded. Complications such as aseptic screw loosening, need for screw replacement, infection, implant removal, neurological injury, and PLRI, among others, were registered and graded according to the Clavien–Dindo–Sink complication classification system [19]. Satisfaction at the last follow-up visit was queried by means of a simple yes/no question.

**Figure 3:**
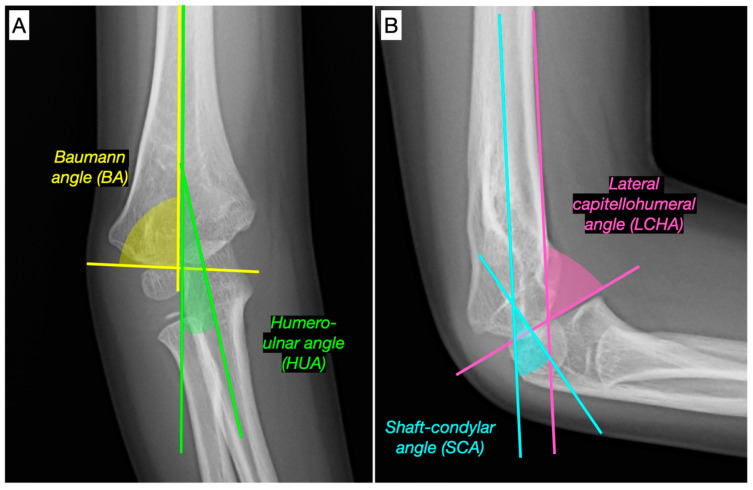
Radiologic measurements on anteroposterior (**A**) and lateral (**B**) elbow radiographs. BA, Baumann angle; HUA, humero-ulnar angle; SCA, shaft-condylar angle; LCHA, lateral capitellohumeral angle.

Clinical CA was measured in the coronal plane using a goniometer in the clinic, with the patient in a standing position and both arms extended on both sides of the body, palms facing forward;while elbow ROM in flexion and extension were measured in the sagittal plane with the elbow in maximum flexion and extension, respectively. The PLRI of the elbow was assessed using the lateral pivot-shift test and the chair rise test. Pre- and postoperative radiologic measurements (Figure 3) included the humero-ulnar angle (HUA), defined as the angle formed between the longitudinal axis of the humerus and the axis of the proximal third ulna (normal 5–15°) [20,21]; the Baumann angle (BA), determined by the humeral axis line and a straight line passing through the capitellar epiphyseal plate (normal values, 64–81°) [22,23]; in the sagittal plane, the shaft-condylar angle (SCA), as the angle between a longitudinal axis of the humerus and a line that bisects the capitellum into equal parts (normal values 40°) [24]; and the lateral capitellohumeral angle (LCHA), defined as the angle between the anterior humeral line and the capitellar physis (normal values 51 ± 6°) [25]. Radiological measurements were performed by 2 experienced pediatric orthopaedic surgeons, who were not involved in the surgical procedures, using the Syngo.plaza PACS software (Siemens Healthcare 2022), and the mean of these measurements was used. Interobserver reliability for the radiologic evaluation was calculated with the Cohen k coefficient, showing a high degree of consistency between the 2 observers, ranging from 0.83 to 0.91.

Data were analyzed with STATA software (Version 16; StataCorp LP, College Station, TX, USA). A descriptive analysis was performed, expressing quantitative variables as median and interquartile range, and qualitative variables as count and percentage. A comparative analysis was also performed between pre- and postoperative clinical and imaging measurements using the Wilcoxon test for paired samples. The Mann–Whitney test for independent samples was used for specific subgroup analyses. Finally, an association analysis was conducted to determine the factors associated with parental satisfaction, degree of correction, and need for screw replacement using the Spearman test. All tests were two-tailed, and statistical significance was set at *p*-values < 0.05.

## 3. Results

### 3.1. Descriptive Analysis

The study cohort comprised 15 patients, 12 of whom were male (80%); the left elbow was affected in 9 (60%) of the patients. The median age at the time of fracture was 3.3 years (IQR, 2.3 to 4). Fractures leading to cubitus varus were 80% supracondylar (12), while 1 patient had a history of a lateral condyle fracture (6.67%), and 2 patients had had more than one fracture over time (13.33%). Management was orthopaedic in 9 cases (60%) and surgical in 6 (40%). The median age at the time of surgery was 5.1 years (4 to 7.1), with a minimum of 3 and a maximum of 10.1 years. Median follow-up was 81 months (64 to 103), with a minimum follow-up of 49 and a maximum of 151 months, while the median implant duration was 72 (63.5 to 89.4) months. The median patient age at the last visit was 13.9 years (10.4 to 15.7), with a minimum of 7 and a maximum of 17.6 years.

### 3.2. Comparative Pre- vs. Post-Analysis

**Figure 4:**
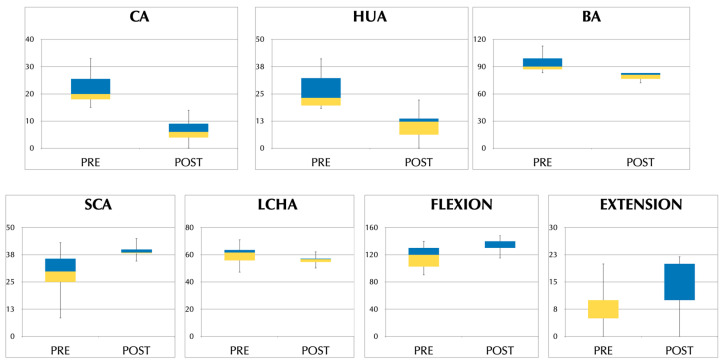
Boxplot representing pre- and postoperative clinical carrying angle (CA), humero-ulnar angle (HUA), Baumann angle (BA), shaft-condylar angle (SCA), lateral capitellohumeral angle (LCHA), and flexion and extension.

Improvement reached statistical significance between the pre- and last postoperative visit for all clinical and radiological variables, as seen in Table 1 and Figure 4; this improvement was more notable for the radiologic measures of HUA, BA, and SCA than for LCHA, as well as being more pronounced for the clinical CA than for the clinical variables of flexion and extension.

**Figure 5:**
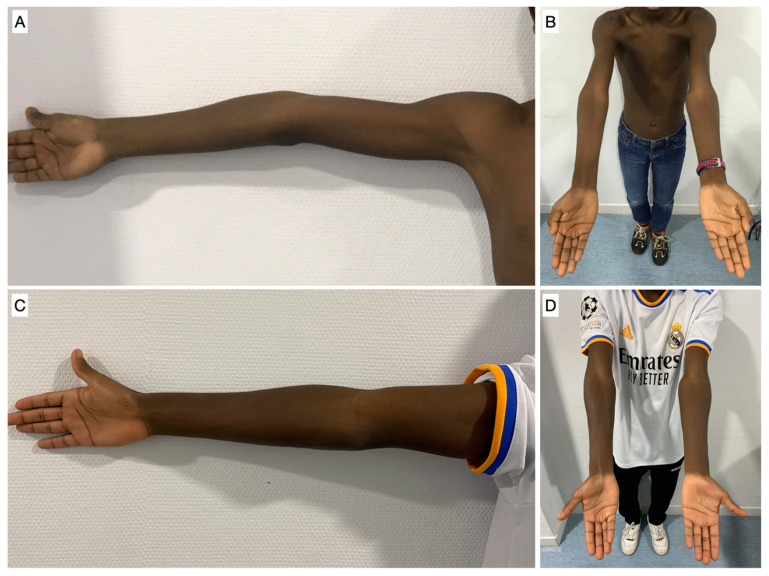
(**A**,**B**) Preoperative clinical images of a patient from the study in which the cubitus varus and hyperextension deformity can be seen. (**C**,**D**) 7-year postoperative clinical images of the same patient in which an improvement of the arm axis can be seen.

**Figure 6:**
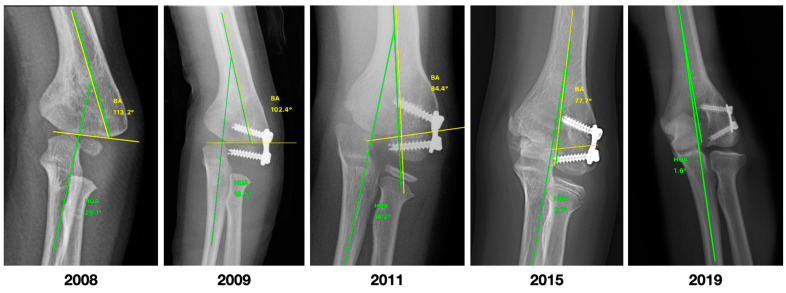
Patient treated with LDHH at the age of 3 years and 5 months, and radiographic images obtained over the following 10 years until the correction of the arm axis. BA, Baumann angle; HUA, humero-ulnar angle.

**Figure 7:**
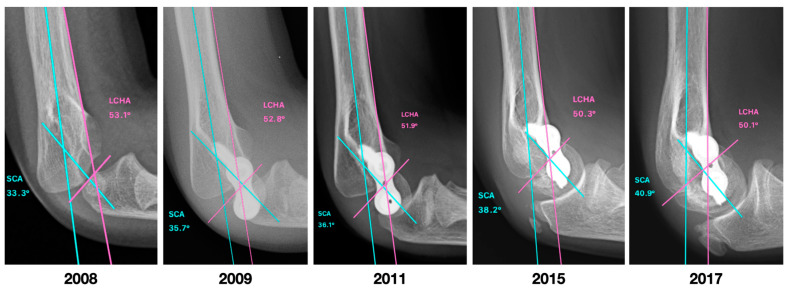
Lateral radiographs of the elbow of the same patient in Figure 5, showing an improvement in the radiological parameters, with values closer to normal in the last radiological control. SCA, shaft-condylar angle, LCHA, lateral capitellohumeral angle.

Figure 5 shows the clinical improvement of a patient in the study, while Figure 6 and Figure 7 show, respectively, the improvement of the coronal and sagittal radiological images of a patient from the study. The correction rate for each measurement was 2.41 (1.9 to 3.2) degrees per year for HUA, 1.51 (1.3 to 2.8) degrees per year for BA, 1.36 (0.5 to 2.1) degrees per year for SCA, and 0.75 (0 to 1.4) degrees per year for LCHA.

### 3.3. Correlation Analysis

**Figure 8:**
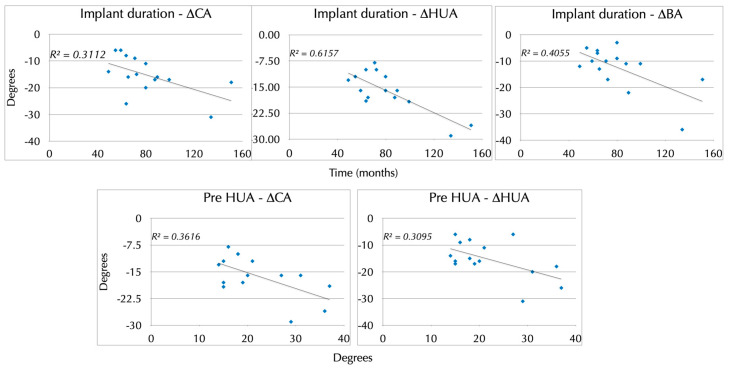
Scatterplot representing the correlation between implant duration and the degree of clinical correction using the CA, and the radiological correction measured by the HUA (*p* = 0.027) and the BA (**upper graphs**). Scatterplot representing the correlation between preoperative HUA in relation to CA degree correction and to HUA degree correction (**lower graphs**). CA, carrying angle; HUA, humero-ulnar angle, BA, Baumann angle.

No correlation was observed between age at the time of intervention and degree of correction in terms of CA (*p* = 0.329), HUA (*p* = 0.281), or BA (*p* = 0.244), nor between age at the time of intervention and the degrees of HUA correction/year (*p* = 0.549) or degrees of BA correction/year (*p* = 0.147). However, a correlation was found between implant duration and the degree of clinical correction using the CA (*p* = 0.007), and the radiological correction measured by the HUA (*p* = 0.027) and the BA (*p* = 0.007). Likewise, a significant correlation was obtained between the previous HUA and the degree of CA correction (*p* = 0.031) as well as with the degree of HUA correction (*p* = 0.040) (Figure 8), meaning that more correction was obtained in patients with a worse previous HUA.

### 3.4. Complications

**Figure 9:**
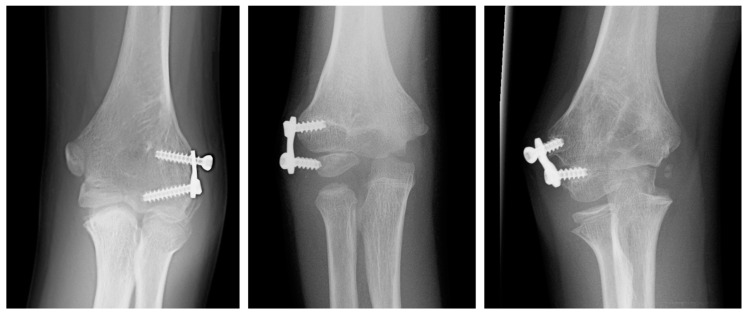
Radiographic images of some of the cases of aseptic loosening due to short screw length and a suboptimal technique in which the screws required replacement with longer and better-positioned screws.

Regarding complications, 6 patients presented at least one complication (40%). Four were classified as Clavien–Dindo–Sink grade III and 2 as grade II. There were 5 cases (33.33%) of aseptic screw loosening. The epiphyseal screw loosened in 2 cases, the metaphyseal screw in 2 others, and both screws in another patient. Four of these patients (26.67%) required replacement; the risk of replacement was not related to age at the time of surgery (replacement 6.31 (2.91) vs. no replacement 4.93 (1.71) years; *p* = 0.324). Following an analysis of the cases, we concluded that these cases of screw loosening were likely to have been the result of a relationship between a short screw length and a suboptimal technique (Figure 9). These screws were replaced with longer ones placed in better position. In addition, there was 1 case of superficial wound infection that resolved with oral antibiotics. Not included as complications, there were 5 cases of implant removal at skeletal maturity or complete correction, at a mean of 79.8 (64.4 to 88.4) months, either at the patient’s request or due to discomfort. None of the children has received a corrective osteotomy to date.

### 3.5. Satisfaction

Most parents or patients (86.67%) were satisfied after the surgery and a relationship was found between parent satisfaction and younger age at surgery, with no relationship found with other variables such as implant duration, initial deformity, or degree of correction (Table 2).

## 4. Discussion

This is the first published series of LDHH using the eight-Plate system as a guided growth technique for the correction of mild–moderate cubitus varus as a sequela of distal humerus fractures in children. In addition to obtaining statistically significant improvements in all clinical and radiological outcomes, the subgroup comparative analysis and the association analysis reveal a number of relevant findings.

There are only three other published papers on guided growth of the lateral distal humerus: two case reports [16,17] and the recent short series of five patients published by Soldado et al. [18] (Table 3). The two case reports use the eight-Plate, as in our study, reporting good results. However, the authors of both studies do not provide convincing radiologic or clinical images showing the correction obtained, and both have a short follow-up time (12 and 18 months). The results from Soldado et al. are unsatisfactory, describing a 1 degree of correction as determined by clinical CA. Although none of the different guided growth techniques has been found to be superior to others [15,26], these disappointing short-term results may be due to insufficient fixation related to the reduced number of screw threads available to secure the small, cartilaginous epiphysis of the lateral humeral condyle with the use of an oblique cannulated screw from the medial side. Another factor behind the limited success reported by these authors may be the short follow-up period (mean of 3 years and 10 months) and the absence of a more accurate method to evaluate the results, such as radiographic measurements, since only clinical assessment was performed. Our study, in contrast, does report improvements in both clinical and radiological coronal and sagittal measures in a larger group of patients and with longer follow-up.

In addition to the clinical and radiographic improvements obtained, other conclusions from our study merit comment. First, many of the patients are still under treatment and, due to their age (5 patients aged under 12 years at the end of the follow-up), have potential for further growth, so it is expected that the deformity will continue to improve. Therefore, although the median CA and HUA at the last measurement remain at 6° and 8° of varus, respectively, we believe that these outcomes will improve with time.

**Figure 10:**
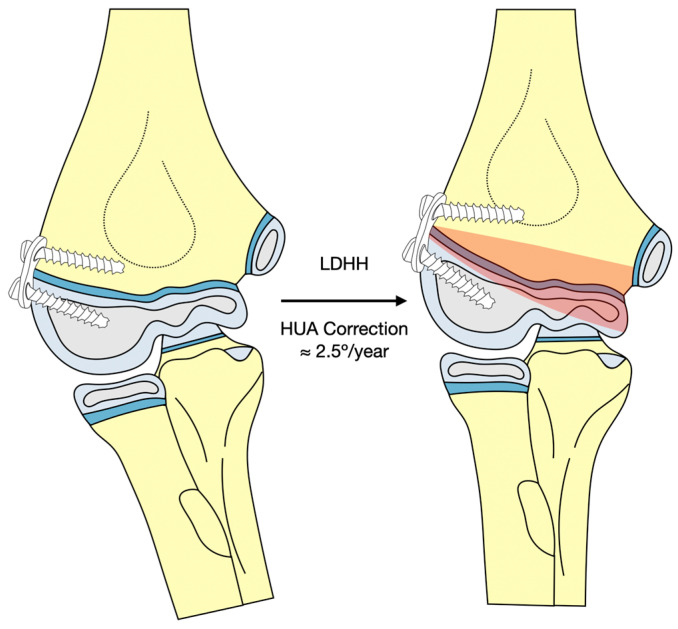
Illustration of the effect of the LDHH technique with the eight-Plate system for cubitus varus correction, with an approximate correction rate of 2.5° for the HUA. LDHH, lateral distal humeral hemiepiphysiodesis; HUA, humeral-ulnar angle.

Despite the suboptimal results published in a recent series [18], we obtained a statistically significant correction of the deformity. It is true, however, that due to the lower growth potential of the distal humerus compared with the other anatomical regions, which accounts for 20% of humerus growth and 10% of limb growth [27,28], the annual correction rate is low, at 2.41° (1.9 to 3.2) for HUA and 1.51° (1.3 to 2.8) when measured by BA. We have not found a correlation between patient age at intervention and the annual correction rate according to the HUA or BA. This suggests a fairly uniform growth rate of the distal humerus during childhood, as this bone is not greatly affected by the growth spurt of early childhood and adolescence. We also found no correlation between age at intervention and degree of correction in terms of clinical CA, HUA, or BA. However, we did find a significant association between the implant duration and the degree of clinical and radiologic correction. These apparently incongruent results point to a need for further follow-up in some young patients, in whom there is still potential for correction. Thus, although implant duration and age at implantation are interrelated, the duration of implant retention is more important than the age at which the patient undergoes the procedure. Knowing the approximate annual correction rate of 2.5° achieved by LDHH with the eight-Plate system estimated by this study (Figure 10), and based on the patient’s age and the severity of the deformity, one can calculate whether this technique will allow complete correction of the deformity over the remaining time of growth. Thus, to correct a 12° deformity to neutral, 5 years are needed (2.41° × 5 = 12°) and for a 17° deformity, 7 years would be needed (2.41° × 7 = 17°), adding 3 more years to achieve a more normal 8 degrees of valgus to each case. Based on this study, we can state that LDHH can be indicated in children from 3 up to 10 years of age, considering that the younger the patient, the greater the potential for correction.

Additionally, the study of the sagittal plane has allowed us to observe a gradual correction in cases of malalignment. Most of these patients with a cubitus varus deformity also presented a certain degree of hyperextension, with a decreased SCA and an increased LCHA. Placement of the eight-Plate in the most anterior area on the condyle in cases of hyperextension might lead the physis to become more vertically oriented and this segment to flex progressively. This improvement in the sagittal plane may also be due to remodeling with growth and elbow motion. In cases where there was no such malalignment in hyperextension, placement of the lateral plate more centered on the condyle allowed the sagittal alignment to be maintained. It should be noted that rotational deformities cannot be corrected with this technique.

Regarding complications, the percentage of aseptic screw loosening observed is noteworthy. As mentioned above, the small size of the epiphysis of the lateral humeral condyle and its cartilaginous nature in young children could explain some of the cases. However, as we found no relationship between age at the time of surgery and the need for replacement due to loosening, together with the fact that some of the loosened screws had been placed in the metaphysis, a critical analysis of the failed cases led us to conclude that the most likely cause was insufficient screw length and failure of the technique. This is probably part of a learning curve on how to implant the plate and screws in this location. We recommend that patients are advised of the relatively long duration of implant retention and the possibility of reoperation for screw replacement, although this risk is minimized with a good technique and adequate screw length. The superficial location of the lateral area of the distal humerus, which offers little soft tissue coverage, can lead to implant-related discomfort and the need for removal at the end of growth or when correction is achieved, which in our series had occurred in 33% of the cases at the time of the study.

We found that all except two patients were satisfied with the treatment, since they experienced gradual correction of the deformity with a minimally invasive method. We found no relationship between satisfaction and implant duration, initial deformity, or degree of correction, and the only significant relationship found was with age at implantation, with the two oldest patients at the time of initial surgery (7.9 and 10.1 years) being dissatisfied, although an adequate axis was achieved in both (4° and 8°, respectively).

This study has certain limitations. The first limitation concerns its retrospective nature and small sample size, despite it being the largest series of LDHH published to date. Secondly, some patients remain in follow-up and have remaining growth potential to improve the arm axis. Therefore, it is likely that more satisfactory outcomes would be reflected with longer follow-up, giving the younger patients adequate time to reach skeletal maturity.

## 5. Conclusions

In conclusion, these results show that LDHH with the eight-Plate system is an effective technique for the correction of mild–moderate cubitus varus deformity in children, and that the approach has greater corrective capacity the longer the implant is in place. The annual correction rate according to HUA of around 2.5° found in this study enables the estimation of whether this technique is suitable for a given patient according to age and the severity of the deformity. In view of the low annual correction rate, long-term studies, such as the one presented here, should be undertaken to evaluate the outcomes, and no robust conclusions should be drawn from short-term studies. Most patients are satisfied after the procedure, with age being a determining factor in these appraisals. Patients should be advised of the relatively long duration of implant retention and the potential need for implant removal at the end of growth, due to discomfort.

## Figures and Tables

**Table 1:** Pre- and post-clinical and radiological measurements.

	PRE	POST	Difference	*p* Value
CA	−20 (−18 to −26)	−6 (−4 to −9)	−16 (−18 to −9)	<0.001
HUA	−19 (−15 to −29)	−8 (−1 to −10)	−16 (−19 to −12)	<0.001
BA	90 (87 to 100)	81 (76 to 83)	−11 (−17 to −7)	<0.001
SCA	29.8 (24.3 to 36.2)	38.5 (38.1 to 40)	7.5 (3.3 to 13.8)	<0.001
LCHA	61.6 (54 to 64.1)	56.7 (54.6 to 57.3)	−4.8 (−6.8 to 0.6)	0.018
Flexion	120 (100 to 130)	130 (130 to 140)	10 (0 to 24)	0.006
Extension	0 (−10 to 0)	0 (o to 10)	10 (0 to 10)	0.013

Values are represented as median (IQR). Comparative study performed with Wilcoxon test for paired samples. CA, carrying angle; HUA, humero-ulnar angle; BA, Baumann angle; SCA, shaft-condylar angle; LCHA, lateral capitellohumeral angle.

**Table 2:** Comparison results between satisfied and unsatisfied patients for different variables.

	Satisfied	Unsatisfied	*p* Value
Age	4.93 (3.9 to 6.7)	9.00 (7.9 to 10.1)	0.037
Implant duration	72.17 (61.3 to 93.3)	76.45 (63.5 to 89.4)	0.466
Initial clinical CA	21 (17.5 to 28)	19 (18 to 20)	0.304
Initial HUA	19 (15 to 30)	19 (18 to 20)	0.5
CA correction	−16 (−19 to −10)	−12 (−16 to −8)	0.248
HUA correction	−16 (−19.1 to −12)	−12 (−15 to −9)	0.196

Values are represented as median (IQR). Comparative study performed with Mann–Whitney test for independent samples. CA, carrying angle; HUA, humero-ulnar angle.

**Table 3:** Summary of published articles on lateral distal humerus hemiepiphysiodesis.

Study	N	Mean Age (y)	Previous Fracture	Pre HUA	Technique	Post HUA	FU (m)
Almahmudi 2020 [16]	1	10	SHF	−3°	Eight-Plate and screws	+6°	12
Verka 2021 [17]	1	5.67	SHF	−22.4°	Eight-Plate and screws	NR	18
Soldado 2022 [18]	5	3.58 (2.5 to 4.42)	NR	−22.5° (−15 to −30) *	Medial oblique cannulated screw	−21° (−15 to −30) *	46
Current study 2023	15	5.1 years (4 to 7.1)	12 SHF, 1 CHF, 2 multiple	−19 (−15 to −29)	Eight-Plate and screws	−8 (−1 to −10)	81 (64 to 103)

y, years; SHF, supracondylar humerus fracture; CHF, lateral condyle humerus fracture; HUA, humero-ulnar angle; FU, follow-up; m, months; NR, Not reported. * Values represent clinical carrying angle.

## Data Availability

The data presented in this study are available on request from the corresponding author.
